# Dr. Barker and the Pauper Insane Again

**Published:** 1892-09

**Authors:** 


### DR. BARKER AND THE PAUPER INSANE AGAIN.

The editorial on this subject in our last issue was not prompted by any animosity to Dr. Barker, nor written with any intention of reflecting upon him unjustly,—far from it;—but for a very different purpose, and from an animus evidently not appreciated.

The JOURNAL commended Dr. Barker's appointment, believing it to be as good a selection as the governor could have made from the ranks of the medical profession of Texas amongst the inexperienced in asylum management; therefore, the JOURNAL not only felt deeply interested in the grave charges brought against him, but was sincerely desirous that he should defend himself. Of course, we do not blame him for taking no notice of the newspaper attacks upon him; but the matter was commented upon so harshly that the JOURNAL felt that Dr. Barker owed it to himself, to his friends and to the Governor to make an explanation,—assuming, of course, that he had *reasons* for his action, which, as we said before, in the absence of explanation looked like an outrage. We have heard much expression of sentiment from medical men,—many of whom, like ourselves, are friends of Dr. Barker, and the Doctor was univerally censured for his action in refusing to receive Purnell into his asylum.

Either Dr. Barker is placed in a false light by the reports on this subject, or he is open to just censure. Believing that the former is the case, we wrote the article in question, in the hope that he would write a reply and defend his action,—at least give his views of his duty in the premises.

While it is not for us to put excuses in his mouth, it was fair to assume—and we did assume—that Dr. Barker had other reasons for refusing to take Purnell than that attributed to him,—sentiment. For our own part, if we were in Dr. Barker's place, we should have felt that a superintendent ought to have some power or right of discrimination in the admission of patients, and if a dangerous paranoiac is to be admitted, we should have felt morally obliged to keep him securely confined, locked up and guarded, and thus rendered harmless; and it may be that there had been no provision made for securely locking up patients of this class and caring for them separately. With regard to Dr. Barker's alleged refusal to receive colored patients, it might be that the board of managers had made no provision for this class separately, and Dr. Barker very properly declined to receive them and mix them with the whites.

But,—to date the Doctor has not seen fit to define his position nor defend his action, and we are disappointed. It would have afforded the JOURNAL great pleasure to publish a letter from him, and if he could justify himself, as we had hoped he would do to back him up. If, as was said before,—he has no explanation to make, and has acted simply from impulse,—if it is a question of like or dislike, and he defies the public sentiment and is indifferent to the construction put upon his action by the profession, he is very justly censurable; it demonstrates his unfitness for the high trust reposed in him and a misplaced confidence on the part of the Governor. We shall still hope the Doctor will see the importance of an explanation, and we again offer the JOURNAL as the proper medium for its publication.

